# Multiethnic Genetic Association Studies Improve Power for Locus Discovery

**DOI:** 10.1371/journal.pone.0012600

**Published:** 2010-09-08

**Authors:** Sara L. Pulit, Benjamin F. Voight, Paul I. W. de Bakker

**Affiliations:** 1 Division of Genetics, Department of Medicine, Brigham and Women's Hospital, Harvard Medical School, Boston, Massachusetts, United States of America; 2 Program in Medical and Population Genetics, Broad Institute of Harvard and Massachusetts Institute of Technology, Cambridge, Massachusetts, United States of America; 3 Department of Medical Genetics, University Medical Center Utrecht, Utrecht, The Netherlands; 4 Julius Center for Health Sciences and Primary Care, University Medical Center Utrecht, Utrecht, The Netherlands; Peninsula Medical School, United Kingdom

## Abstract

To date, genome-wide association studies have focused almost exclusively on populations of European ancestry. These studies continue with the advent of next-generation sequencing, designed to systematically catalog and test low-frequency variation for a role in disease. A complementary approach would be to focus further efforts on cohorts of multiple ethnicities. This leverages the idea that population genetic drift may have elevated some variants to higher allele frequency in different populations, boosting statistical power to detect an association. Based on empirical allele frequency distributions from eleven populations represented in HapMap Phase 3 and the 1000 Genomes Project, we simulate a range of genetic models to quantify the power of association studies in multiple ethnicities relative to studies that exclusively focus on samples of European ancestry. In each of these simulations, a first phase of GWAS in exclusively European samples is followed by a second GWAS phase in any of the other populations (including a multiethnic design). We find that nontrivial power gains can be achieved by conducting future whole-genome studies in worldwide populations, where, in particular, African populations contribute the largest relative power gains for low-frequency alleles (<5%) of moderate effect that suffer from low power in samples of European descent. Our results emphasize the importance of broadening genetic studies to worldwide populations to ensure efficient discovery of genetic loci contributing to phenotypic trait variability, especially for those traits for which large numbers of samples of European ancestry have already been collected and tested.

## Introduction

Over the past four years, genome-wide association studies (GWAS) have started to reveal the genetic underpinnings of complex traits and common diseases, yet only a modest fraction of the heritability can be attributed to the collection of associated variants discovered to date [1]. Even though impressive sample sizes have been assembled through collaborative efforts, the statistical power to discover susceptibility loci is limited by a number of factors [2].

One limitation of the first wave of GWAS is the almost exclusive interrogation of common variation with limited coverage of alleles in the lower end of the frequency spectrum. Many low-frequency alleles have not been ascertained, and even for those that were catalogued in the dbSNP database, SNPs on the genome-wide microarrays tag low-frequency variants only poorly through pairwise linkage disequilibrium [3]. The second limitation is that most GWAS to date have primarily studied samples of European descent [4], with low power to detect association for alleles of low frequency compared to more common alleles.

A compelling illustration of this second limitation was provided by the recent discovery of the association at the *KCNQ1* locus with type 2 diabetes risk (with an estimated odds ratio  = 1.2) in two contemporary GWAS in East-Asian population samples [5], [6]. The associated SNP (rs2283228) has a minor allele frequency of ∼40% in East-Asian samples but a minor allele frequency of ∼5% in European samples [7]. At this allele frequency, the association had little power to be discovered (at p<5×10^−8^) in the series of GWAS in European-derived samples performed ahead of the two East-Asian studies [8], [9], [10], [11], [12], even though the SNP was well tagged in European samples. After its initial discovery in the East-Asian samples, the association was successfully replicated as a pre-specified hypothesis at a more liberal significance threshold. This finding raises the question to what extent power is affected by the focus on samples of European ancestry, and how power to detect association of genetic variation segregating at low frequency in European populations could be heightened by broadening GWAS efforts to a more diverse set of populations.

To this end, we evaluate here the relative benefit of performing future genome studies in worldwide populations (“second phase”), preceded by an initial GWAS in a European population (“first phase”). Using the empirically observed allele frequencies in population samples represented by HapMap Phase 3 and the 1000 Genomes Project, we quantify the impact of frequency differences between populations on the power to find novel association of modest effect (GRR ≤1.5), assuming that genome-wide association results are combined in the two GWAS phases. We also address the implications of such allele frequency differences for replicating *bona fide* associations (most discovered in European samples) in different populations.

Overall, we show that there are substantial power gains to be had by focusing on large multiethnic studies. Additionally, allele frequency fluctuations between global populations and their impact on power must be considered in replication studies.

## Methods

We computed power to detect an association at genome-wide significance (p<5×10^−8^) using a theoretical model of log-additive (multiplicative) effects, allowing us to perform all calculations efficiently. For each SNP, we separately computed the non-centrality χ^2^ parameter (NCP) in the first and second phase as a function of risk allele frequency, case-control sample size, and assumed effect size (genotype relative risk, GRR) (**Text S1**). In phase 1, we used the empirically observed allele frequencies in CEU (Utah residents with Northern and Western European ancestry collected by the Centre d'Etude du Polymorphisme Humain) to mimic the fact that most GWAS to date have tested European samples. In phase 2, we used the allele frequencies in any of the population panels represented in HapMap 3 or 1000 Genomes (see below). For multiethnic scenarios in phase 2, we computed the NCPs per population. By summing the NCPs in all populations in phases 1 and 2, we derived the asymptotic power for a given SNP to reach a p-value of 5×10^−8^ (**Text S1**). For each SNP, we averaged the power for both alleles simulated as the risk-increasing allele (GRR >1). We repeated this procedure across all 1,440,616 SNPs present in HapMap 3 or 3,327,757 SNPs present in 1000 Genomes, and report the average genome-wide power across all SNPs, and the average power for SNP subsets stratified by the minor allele frequency in CEU (where we refer to 1–5% as low-frequency alleles). We varied the total sample size of each scenario between 10,000, 20,000 and 80,000 samples (**Table 1**). We varied the GRR between 1.1 and 1.5, following a multiplicative risk model and assumed a fixed effect between different populations in phases 1 and 2.

**Table 1:** Overview of the three main GWAS scenarios.

Total Sample Size	Phase 1		Phase 2	
	Cases/controls	HapMap panel	Cases/controls	HapMap panel
10,000	2,500/2,500	CEU	2,500/2,500	CEU, TSI, CHB, CHD, JPT, YRI, MKK, LWK, ASW, MXL, or GIH
20,000	5,000/5,000	CEU	5,000/5,000	CEU, TSI, CHB, CHD, JPT, YRI, MKK, LWK, ASW, MXL, or GIH
80,000	10,000/10,000	CEU	30,000/30,000	CEU, TSI, CHB, CHD, JPT, YRI, MKK, LWK, ASW, MXL, or GIH, or CEU+CHB+YRI or CEU+CHB+YRI+MXL+GIH+ASW or CEU+CHB+YRI+MXL+GIH+ASW

We note that our approach is not to be confused with a two-stage replication design (as described, for example, in [13]) where only a limited set of SNPs are taken from the phase 1 (based on some p-value threshold) for additional testing in phase 2. Instead, we leverage the idea that genome-wide association data sets are combined in collaborative spirit, as is now routinely done for locus discovery.

We obtain empirical allele frequencies for SNPs from two sources: HapMap 3 and the 1000 Genomes Project. The HapMap 3 resource comprises genome-wide SNP data from 11 population samples [14]: CEU (Utah residents with Northern and Western European ancestry collected by the Centre d'Etude du Polymorphisme Humain), TSI (Toscans in Italy), CHB (Han Chinese in Beijing, China), JPT (Japanese from Tokyo, Japan), CHD (Chinese in Metropolitan Denver, Colorado), YRI (Yoruba in Ibidan, Nigeria), MKK (Massai in Kinyawa, Kenya), LWK (Luhya in Webuye, Kenya), ASW (African ancestry in southwest USA), GIH (Gujarati Indians in Houston, Texas), and MXL (Mexican ancestry in Los Angeles, California). In total, 1184 samples have been genotyped on the Illumina Human-1M and Affymetrix 6.0 arrays, and SNP genotypes merged and processed for quality control. Our analysis is restricted to the allele frequencies of 1,440,616 SNPs that are QC-passing and polymorphic in at least one of the eleven populations (referred to as the consensus data). To remove bias due to different sample sizes of these populations, we randomly selected 50 unrelated founders (100 unique chromosomes, dictated by MXL, the smallest sample) from each population in the consensus genotype data to compute unbiased allele frequencies.

We also used frequency estimates from low-coverage sequencing data in four population panels (CEU, CHB, JPT, and YRI) generated in Pilot 1 of the 1000 Genomes Project [15]. The data we use here was generated in 60 founder individuals of CEU, 30 of CHB, 30 of JPT, and 59 of YRI, resulting in three analysis panels of similar sample size (CEU, CHB+JPT and YRI).

As a complementary analysis, we performed a power analysis for a specific set of 182 unique SNPs (189 reported associations; 7 SNPs reported for multiple diseases) that have recently been described as significantly associated to 26 complex diseases (taken from http://www.genome.gov/GWAStudies/, accessed on 28 September 2009). For most of these associations, the effect sizes range from 1.1 to 1.3. Based on the observed allele frequencies in each of the HapMap 3 populations, we computed the sample size required to replicate the association at 80% power (at a nominal p<0.05) for the reported risk allele, taking the reported odds ratio as the GRR in a multiplicative model. This analysis reflects the testing of a specific hypothesis (instead of discovery across the whole genome) based on *bona fide* associations reported by GWAS performed to date.

## Results

We compared three main scenarios where the key difference was the total sample size (**Table 1**). First we explored the simple scenario where phases 1 and 2 are performed in 5,000 samples from the CEU population panel (**Table S1**). Power was, as expected, consistently lower across the entire range of effect sizes and allele frequencies compared to a sample size of 20,000 (**Table S2**) and 80,000 (**Table S3**).

At a sample size of 20,000, the aggregate power across all 1.4 million SNPs considered was 82% at an effect size (GRR) of 1.5, but fell to 8% at a GRR of 1.1 (**Table S2**). Across the different effect sizes, power was largely driven by the power contribution of common alleles (MAF >10%). At a modest GRR of 1.2, the power of common alleles was 87% and rose to nearly 100% at a GRR of 1.5. For lower frequency variants (MAF ≤5%), power to detect an association remained low. For a GRR of 1.1 or 1.2, power for SNPs with 1–5% frequency was essentially zero, but rose to 65% when GRR was 1.5. Increasing the effect size reduced the minimum allele frequency at which an association could be detected. For a variant of 3% frequency, there was virtually no power to detect at an effect size of 1.2 but almost 60% power to detect it at a GRR of 1.4.

For a sample size of 80,000 samples, power to detect an association of a lower frequency allele (MAF ≤5%) remained low at smaller effect sizes. Power is ∼3% at GRR = 1.1 (but ∼89% for common variants with the same effect size), and did not rise above the 80% power threshold until a GRR of 1.3 (**Table S3**).

Next we compared the two European samples (CEU and TSI) in phase 2 and found that the aggregate estimates for both populations were very similar for all scenarios considered. A power gain in TSI could be observed for lower-frequency variants at smaller total sample sizes (10,000 and 20,000), including variants that appear monomorphic in CEU (which by definition cannot contribute power). This effect, however, was balanced by a power loss in TSI for the largest sample size (80,000), due to the total number of monomorphic SNPs in TSI (241,688 SNPs compared to 199,821 monomorphic SNPs in CEU; **Figure S1**) and the amplification of their inability to contribute power by the large number of samples.

**Figure 1:**
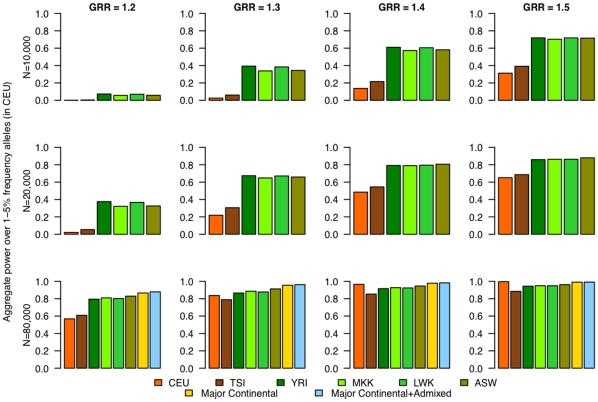
Power to detect association for lower-frequency alleles (≤5%) in CEU based on HapMap 3 data. Power is given for various individual population panels (CEU, TSI, YRI, MKK, LWK, and ASW), a panel with major continental representation (CEU+CHB+YRI) and a cosmopolitan panel with major continental representation and admixed populations (GIH, MXL, and ASW) interrogated in phase 2, aggregated over those alleles that have lower frequency (1–5%) in CEU.

Performing phase 2 of the GWAS in a non-European population panel demonstrated a marked power gain for alleles with low frequency in CEU (MAF ≤5%), in a trend that remained true with increasing sample size or greater effect sizes (**Figure 1** and **Figure S2** for alleles of 5–10% frequency in CEU). Any of the African population panels (YRI, MKK, or LWK) and the admixed African-American (ASW) panel provided the greatest power gain as compared to a GWAS performed in CEU samples. For a sample size of 20,000, the power to detect low-frequency variants (MAF ≤5%) with a GRR of 1.2 was close to zero in CEU, but about 40% in YRI. At a GRR of 1.4, power was about 50% in CEU but 80% in the African population samples. These results demonstrate that there is a notable gain in power for this class of variants by moving into an African population sample for phase 2.

Power for alleles of low frequency in CEU also increased by performing phase 2 in samples from one of the East-Asian populations (CHB, CHD, or JPT) though the gain was not as significant as that provided by moving to an African population sample or a sample from the admixed ASW panel in phase 2. The other admixed population panels (GIH, MXL) also improved power, but to a lesser degree than one of the East-Asian panels.

**Figure 2:**
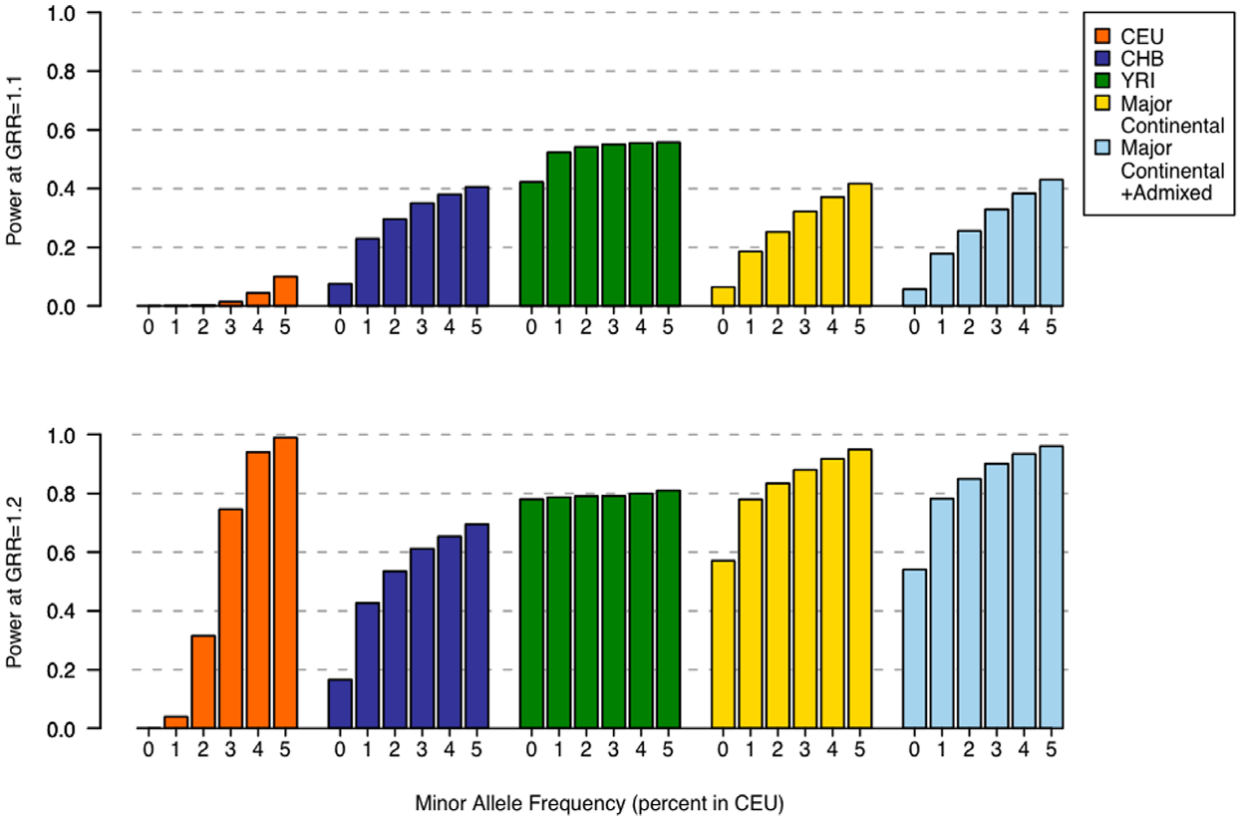
Power as a function of allele frequency (≤5%) in CEU based on HapMap 3 data. For a sample size of 80,000 and a modest effect size (GRR of 1.1 and 1.2), power is given for CEU, CHB, YRI, and two multiethnic panels (“major continental”, CEU+CHB+YRI, and “cosmopolitan”, CEU+CHB+YRI+ASW+GIH+MXL) in phase 2. Including non-European samples in phase 2 improves power to detect an association for alleles that have lower frequency in CEU.

For a sample size of 80,000, performing phase 2 of the GWAS in a non-European population sample continued to yield a net gain in power, but only for those alleles that have low frequency in CEU and for smaller effect sizes (**Figure 2**). For larger effect sizes (GRR >1.3), polymorphic SNPs in CEU (especially those at lower frequency where power can still be improved) continued to gain in power, while the power for many of the other populations reached a plateau due to a comparatively larger slice of apparently monomorphic SNPs (**Figure S1**), an effect due to ascertainment bias of the SNP genotyping platforms used in HapMap 3.

We also examined the utility of two different multiethnic samples in phase 2. One multiethnic sample comprised the major continental population panels (CEU+YRI+CHB) while the second comprised the major continental panels in addition to the admixed panels (CEU+YRI+CHB+GIH+MXL+ASW). Although the power gain for alleles of lower frequency in CEU was noticeable in both multiethnic panels, the best power was obtained for the “cosmopolitan” panel with representation from the major continental groups as well as the admixed populations (**Figures 1** and **2**).

During the course of this study, we had access to low-coverage pilot data from the 1000 Genomes Project, which allowed us to replicate these relationships across 3,327,757 detected variable sites with estimated allele frequencies in the CEU, CHB, JPT and YRI panels across the whole genome, essentially free of ascertainment bias. Overall, the power estimates based on these allele frequency distributions were unchanged (**Tables S4, S5 and S6**), compared to the HapMap 3-based results, also for a lowered discovery threshold of p<1×10^−8^ to reflect increased variation in African samples (data not shown).

**Figure 3:**
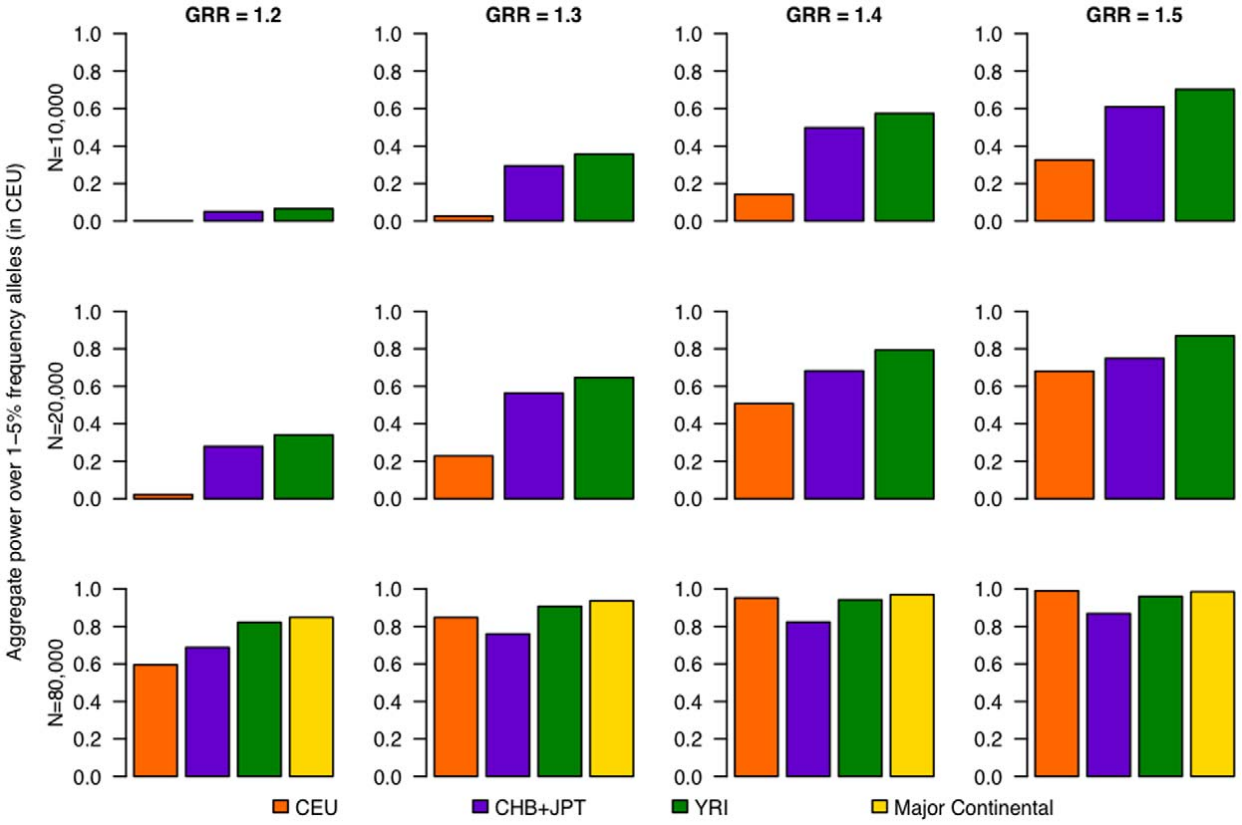
Power to detect association for lower-frequency alleles (≤5%) in CEU using 1000 Genomes Project data. Power is given for three individual panels (CEU, CHB+JPT, YRI), and a multiethnic panel (CEU+CHB+JPT+YRI) in phase 2, aggregated over those alleles that have lower frequency (1–5%) in CEU.

**Figure 4:**
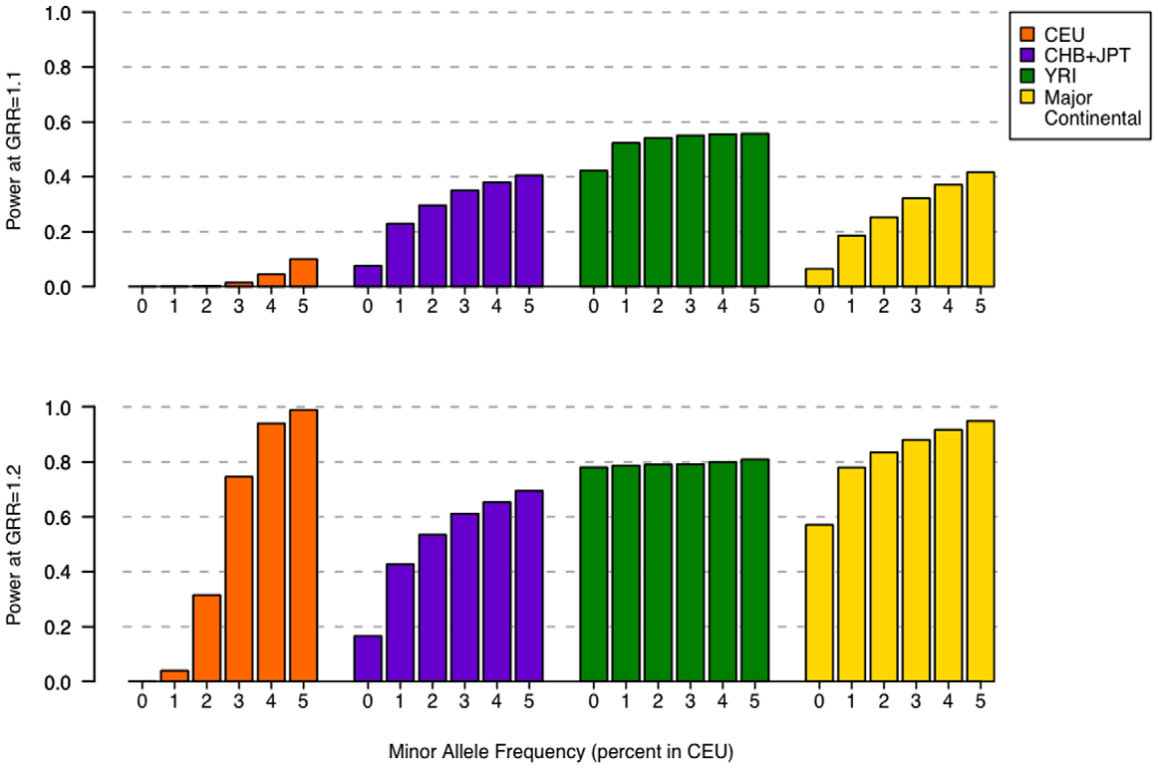
Power as a function of allele frequency (≤5%) in CEU using 1000 Genomes Project data. For a sample size of 80,000 and modest effect size (GRR of 1.1 and 1.2), power is given for three individual panels (CEU, CHB+JPT, YRI) and a multiethnic panel in phase 2. Including non-European samples in phase 2 improves power to detect an association for alleles that have lower frequency in CEU.

Consistent with the HapMap 3-based results, power to detect an association was driven primarily by common variation and power to detect lower-frequency variants remained limited for GWAS scenarios exclusively in European samples (**Tables S4, S5 and S6**). For lower-frequency variants (MAF ≤5%), the 80% power threshold was not reached until the sample size was 80,000 and the effect size was 1.3 (**Figure 3**), exactly as was observed in the HapMap 3 dataset. Performing phase 2 in African ancestry samples increased power to detect alleles of lower frequency in CEU (**Figure 4** and **Figure S3** for alleles of 5–10% frequency in CEU). Power was also increased by including samples of East-Asian ancestry in phase 2, but to a lesser extent than samples of African ancestry. Following a multiethnic approach by combining samples from the CEU, CHB, JPT and YRI panels, we see again a power improvement for lower frequency alleles (in CEU). At an effect size of 1.2 and a total sample size of 80,000, utilizing the multiethnic approach in phase 2 increased power to detect low-frequency variants in CEU to 85%, in comparison to 60% power when only European samples are tested in phase 2. These results indicate a potential gain in power to detect alleles of lower frequency in European samples by performing a second phase of GWAS in samples of African descent (**Figures 3** **and** **4**).

**Figure 5:**
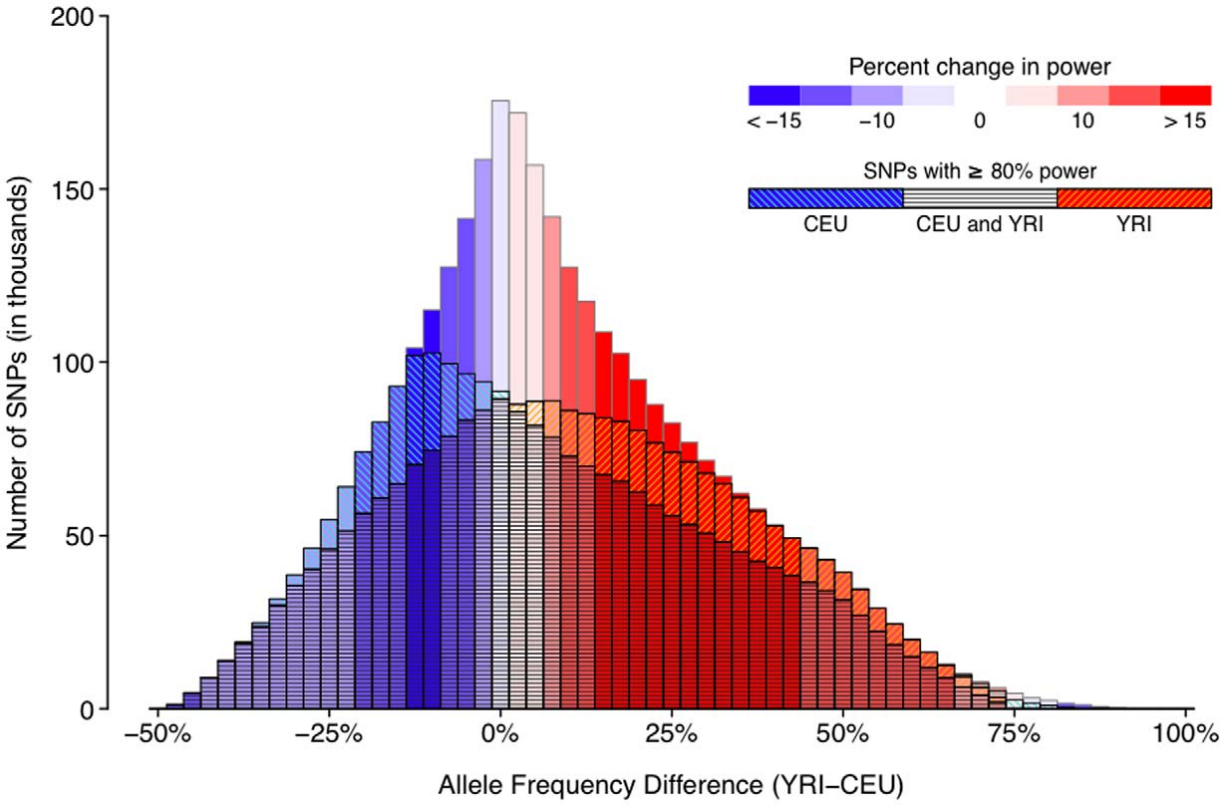
The relationship between allele frequency differences between CEU and YRI and power. We plot the histogram of all SNPs in the 1000 Genomes Project data as a function the allele frequency difference between CEU and YRI (excluding SNPs monomorphic in both CEU and YRI). The histogram is colour-coded by the estimated change in power by performing phase 2 in YRI instead of CEU, assuming a total sample size of 20,000 (10,000 in CEU in phase 1, and 10,000 in YRI in phase 2) and a GRR of 1.2. Allele frequency differences from +15% to +40% in YRI result in a positive gain in power (in red), which is compensated by SNPs that are common in CEU (in blue). We divide the histogram into 4 categories: (1) SNPs with at least 80% power in both scenarios (CEU in phase 2 or YRI in phase 2) (65.6% of all SNPs considered), (2) SNPs with at least 80% power to detect an association in the European GWAS (CEU in phase 2) (6.5% of all SNPs considered), (3) SNPs with at least 80% power in the African GWAS (YRI in phase 2) (9.3%), and (4) SNPs that do not reach 80% in either of these two scenarios (18.6%). As alleles of higher frequency in CEU are mostly saturated for power, including additional European samples in GWAS will only marginally increase power, whereas alleles of lower frequency in CEU may substantially benefit in terms of power from elevated frequencies in African populations.

To illustrate the impact of allele frequency differences between populations, we focus on two specific scenarios: one GWAS in which phase 1 and phase 2 are performed in 10,000 European (CEU) samples (20,000 total samples), and a second GWAS in which phase 1 tests 10,000 European (CEU) samples and phase 2 tests 10,000 African (YRI) samples (**Figure 5**). Of all >3 million SNPs with allele frequency estimates in CEU and YRI from 1000 Genomes, we can identify four categories: (1) SNPs that reached power ≥80% in both scenarios (65.6% of all >3 million SNPs), (2) SNPs with power ≥80% in only the European GWAS (6.5%), (3) SNPs with power ≥80% in only the GWAS with YRI samples in phase 2 (9.3%), and (4) SNPs that do not reach 80% power in either scenario (18.6%). Most of the power gain (3.3%) for the CEU+YRI design (relative to CEU+CEU) is due to alleles that are 15–40% higher in frequency in YRI compared to CEU. For these alleles that are 15–40% more common in YRI, the net gain in power relative to the European GWAS is 24.2%. Power for alleles of high frequency in CEU is saturated with little room for improvement; including more European samples will contribute only limited additional power. Power for alleles of lower frequency in CEU is certainly not saturated, and including samples of African descent in phase 2 results in a marked power gain for those polymorphisms that are relatively common in the African population. The gain in power resulting from using samples of African descent in phase 2 appears to be largely driven by alleles that have higher frequency in African samples than in European samples (**Table 2**).

**Table 2:** Number of SNPs with higher or lower minor allele frequency in YRI or CHB+JPT, as compared to CEU in Pilot 1 of 1000 Genomes.

Allele Frequency Difference	YRI (% of total SNPs)	CHB+JPT (% of total SNPs)
Less common in CEU	1499386 (45.06%)	1518752 (45.64%)
Equal to CEU	14321 (0.43%)	93843 (2.82%)
More common in CEU	1814051 (54.51%)	1715163 (51.54%)

We have ignored allele flips (where the minor allele in CEU is the major allele in YRI or CHB+JPT), in order to estimate how many SNPs would be expected to have better power in YRI or CHB+JPT (due to a higher minor allele frequency).

Lastly, we tested how allele frequency differences between populations could influence the power (or sample size required) for replicating a reported association (**Table S7**). For 182 (unique) SNPs previously described as *bona fide* associations discovered in a European sample, we found that 57 SNPs (30% of the reported associations) required a total sample size of at least 2,500 (with a case/control ratio of 1∶1) to replicate the observed effect at 80% power in another European population sample. Performing replication studies in a non-European population may change the required sample size, reflecting allele frequency changes between populations (assuming a constant effect size). For 90 reported associations (48%), a potential efficiency gain can be achieved, as the effect could be replicated in a non-European population with a smaller sample size than that required in a European population. On the other hand, for 42 (22%) of the reported associations, the sample size required for replication in a non-European population was greater than that required for replication in a European population.

## Discussion

In this work, we have performed a quantitative analysis of how allele frequency differences between populations affect the power of genome-wide association studies, conditional on a first wave of GWAS performed strictly in European samples. Probably the most important observation is that genome-wide studies in a diverse set of population samples can offer improved power for discovery as compared to a study that exclusively focuses on European population samples, and that this effect is dominated by those alleles at lower frequency in European populations. Given the investment made to date in GWAS of European samples, the implication of this result is that a second, future wave of genomic association studies would seem to benefit from broad inclusion of non-European samples.

Our approach has limitations, however, and makes some assumptions about the underlying genetic model that are worth highlighting here. First, HapMap examines a biased set of SNPs [16]. The HapMap 3 data set used here is limited to SNPs on the Illumina Human-1M and Affymetrix 6.0 arrays. Both platforms have a strong bias towards polymorphisms of high frequency in European populations, and provide rather poor representation of low-frequency variants or polymorphisms unique to non-European populations. No explicit attempt was made to reconstruct (in an unbiased way) the full allele frequency spectrum in these populations (see, for example, [17]). Our initial aim was simply to mimic the situation in which these arrays would be used in an actual genome-wide study in any of the populations represented by the HapMap 3 collection. We argue that our results for these SNPs are relevant and realistic, since these arrays are still today state-of-the-art in terms of their SNP density and coverage (though likely not for long). Importantly, the results we obtain with the 1000 Genomes Project pilot data directly address this concern. Whole-genome sequencing resulted in near-complete ascertainment of 3,327,757 variants in the same HapMap samples, essentially free of ascertainment bias. Our calculations based on these variants in the four HapMap panels still indicate that a multiethnic approach for future disease studies is going to improve power to discover novel susceptibility loci.

Second, we limited the HapMap 3 analysis to only 100 founder chromosomes in each of the populations to ensure identical precision in the allele frequency estimates of the respective populations. Consequently, we are unable to simulate power for lower-frequency or rare variants. The rapid advances in DNA sequencing platforms will help build the tools to query those variants in the near future in a comprehensive fashion, mitigating some of the shortcomings of HapMap-inspired SNP arrays. In the absence of empirical data on lower-frequency variants in this study, however, we predict that a multiethnic approach will also be beneficial for the study of rare variation, given that rare variation is more likely to be population-specific.

Third, we have made no attempt to model the LD between the (hidden) causal variant and the tested SNPs. For many susceptibility loci, the genetic architecture of risk alleles remains unknown despite ferocious efforts to fine-map the genetic culprits. For the sake of simplicity, our aim was to simulate the observed behavior of associations at common (tag) SNPs consistent with validated associations recorded to date, regardless of the constellation of causal variants underlying them. In a scenario where a tag SNP captures the causal variant in a consistent manner across multiple populations, this approximation would not be expected to change the result. However, if the LD structure between the tag SNP and the causal variant is markedly different between populations, this could lead to a reduction of power. An extreme example is what others have referred to as the “flip-flop” phenomenon, where different populations can show opposite directions of the effect at the tag SNP (that is, risk in one population and protective in another) [18], [19]. Although it is at present unclear how widespread this phenomenon is, we emphasize that this concern will be alleviated when all polymorphisms can be directly tested (either through complete sequencing or through whole-genome imputation). Either way, sequencing data, such as that generated by the 1000 Genomes Project, will help mitigate concerns about underlying LD patterns. The results based on the 1000 Genomes Project data (with much more complete representation of variation across the genome) clearly indicate that, as long as the effect is shared across populations, using samples from non-European populations can yield a gain in power for discovery. The general agreement with the 1000 Genomes results also indicates that the ascertainment bias of the HapMap 3 data was not a significant limitation, at least for the purposes of this analysis.

Fourth, the disease model employed assumes that the effect size is constant between populations. In other words, we have not specifically modeled biological heterogeneity of the allelic effect, or accounted for phenotype differences (including incidence rate or prevalence) between populations. It remains to be seen to what extent the effects of causal variants are variable between populations, but so far the evidence for common polymorphisms seems to suggest that heterogeneity is limited with reproducible effect sizes between populations (including the *KCNQ1* example that motivated this study), even if for most of the *bona fide* loci we still do not know the causal variants driving the effect. Under a model of biological heterogeneity for a given locus, we expect the power benefit to evaporate only in the scenario that the causal effect is only present in specific populations, or in the more extreme scenario that the effect is even reversed.

Lastly, we have not dealt with the practical problem of population stratification, where the test statistic can be inflated due to intrinsic allele frequency differences between cases and controls (due to some bias in ancestry in both groups). Our results are based on ideal conditions where cases and controls are, by definition, sampled from the same population. Yet, it is worth pointing out the potential pitfalls of not correcting for population structure in association analyses [20].

We have focused only on the discovery of novel loci, not on fine-mapping causal variants within already established loci. The localization of the causal variants is a different question beyond the scope of this analysis, but a multiethnic approach will likely be helpful for that purpose as well, as linkage disequilibrium differences between populations can be exploited to narrow the genomic window that harbors the causal variant [21], [22]. Altogether, this analysis gives a robust indication of the potential benefit of performing whole-genome association studies in a diverse set of population samples, a strategy that has thus far been underutilized [4]. This benefit can be expected for data sets acquired using SNP arrays as well as data from sequencing.

## Supporting Information

Text S1 R script for computing power.
(0.03 MB DOC)

Figure S1 Number of monomorphic SNPs in the HapMap 3 population panels. The number of monomorphic SNPs in each population panel are displayed, stratified by minor allele frequency in CEU.
(1.94 MB TIF)

Figure S2 Power to detect an association for common alleles (5–10%) based on HapMap 3 data. The impact on power of switching to non-European samples in stage 2 of the GWAS is limited primarily to alleles of lower frequency in CEU. Testing non-European samples for alleles of common frequency in CEU yields a small (or no) increase in power.
(1.93 MB TIF)

Figure S3 Power to detect an association for common alleles (5–10%) based on 1000 Genomes data. Consistent with our observations in the HapMap data, the improvement in power achieved by using non-European samples is limited to alleles of lower frequency in CEU.
(1.93 MB TIF)

Table S1 Power to detect an association for a sample size of 10,000 using HapMap 3 data. Power is aggregated over all 1.4 million SNPs, over lower-frequency (1–5%) alleles, and over subsets of SNPs stratified by minor allele frequency in CEU. Calculations were made using allele frequencies from all 11 HapMap 3 population panels. GRR is varied between 1.1 and 1.5. Data points referred to in the paper and in figures appear in bold.
(0.03 MB XLS)

Table S2 Power to detect an association for a sample size of 20,000 using HapMap 3 data. Calculations were made using HapMap 3 data. Data points referred to in the paper and in figures appear in bold.
(0.03 MB XLS)

Table S3 Power to detect an association for a sample size of 80,000 using HapMap 3 data. Power is calculated for individual population panels as well as multiethnic GWAS, in which samples for stage 2 of the GWAS are drawn from the major continental population panels or the major continental plus the admixture population panels. Data points referred to in the paper and in figures appear in bold.
(0.04 MB XLS)

Table S4 Power to detect an association for a sample size of 10,000 using 1000 Genomes data. Population panels in the 1000 Genomes data are CEU, CHB+JPT, and YRI. Data points referred to in the paper and in figures appear in bold.
(0.02 MB XLS)

Table S5 Power to detect an association for a sample size of 20,000 using 1000 Genomes data. Data points referred to in the paper and in figures appear in bold.
(0.02 MB XLS)

Table S6 Power to detect an association for a sample size of 80,000 using 1000 Genomes data. Power for individual population panels as well as multiethnic GWAS is calculated for this sample size, as in the HapMap 3 analysis. Data points referred to in the paper and in figures appear in bold.
(0.03 MB XLS)

Table S7 Sample size needed to replicate known GWAS findings in samples from HapMap 3 population panels. The sample size needed to replicate a GWAS finding at ≥80% power is reported for each of the 11 HapMap 3 population panels. Sample sizes listed as “NA” are annotated as such because the particular SNP is monomorphic in that population panel. SNPs that do not reach 80% power for 150,000 samples are footnoted and power at 150,000 samples is given.
(0.06 MB XLS)
