# Supplementary material for: Multiethnic Genetic Association Studies Improve Power for Locus Discovery
Source: PLoS One. 2010 Sep 8;5(9):e12600. doi: 10.1371/journal.pone.0012600 (PMC2935880; doi:10.1371/journal.pone.0012600)
Supplement: Text S1 — R script for computing power. (0.03 MB DOC) [file pone.0012600.s011.doc]

#

# this function computes the non-centrality parameter,

# given the following parameters:

#

# fA = risk allele frequency

# k = prevalence

# rAa = genotype relative risk (Aa)

# rAA = genotype relative risk (AA)

# n_case = number of cases

# n_control = number of controls

#

#

cc_gpc <- function( fA, k, rAa, rAA, n_case, n_control, verbose )

{

# frequency of the non-risk allele

fa <- 1 - fA

# genotype frequencies (per Hardy-Weinberg)

fAA <- fA * fA

fAa <- 2 * fA * fa

faa <- fa * fa

# baseline risk for the aa genotype

raa <- k / ( fAA*rAA + fAa*rAa + faa )

# risk for the genotypes

rrAA <- raa * rAA

rrAa <- raa * rAa

rraa <- raa

# non-risk

nrrAA <- 1 - rrAA

nrrAa <- 1 - rrAa

nrraa <- 1 - rraa

# odds ratio for the 2 genotypes relative to aa genotype

orAa <- ( rrAa / nrrAa ) / ( rraa / nrraa )

orAA <- ( rrAA / nrrAA ) / ( rraa / nrraa )

# genotype frequencies in cases

case_AA <- fAA * rrAA

case_Aa <- fAa * rrAa

case_aa <- faa * rraa

case_sum <- case_AA + case_Aa + case_aa

case_AA <- case_AA / case_sum

case_Aa <- case_Aa / case_sum

case_aa <- case_aa / case_sum

# genotype frequencies in controls

control_AA <- fAA * nrrAA

control_Aa <- fAa * nrrAa

control_aa <- faa * nrraa

control_sum <- control_AA + control_Aa + control_aa

control_AA <- control_AA / control_sum

control_Aa <- control_Aa / control_sum

control_aa <- control_aa / control_sum

# allele frequencies in cases and controls

case_A <- case_AA + case_Aa / 2

control_A <- control_AA + control_Aa / 2

# convert into case-control counts

n_case_A <- 2 * n_case * case_A

n_case_a <- 2 * n_case * ( 1 - case_A )

n_control_A <- 2 * n_control * control_A

n_control_a <- 2 * n_control * ( 1 - control_A )

# 2x2 chi-square test for association

x2 <- chisq.test(matrix(c(n_case_A, n_control_A,

n_case_a, n_control_a), nrow=2, ncol=2),

correct=F)

ncp <- x2$statistic

# return the non-centrality parameter

ncp

}

#

# this function computes power based on:

#

# ncp1 = ncp of association study in wave 1

# ncp2 = ncp of association study (or studies) in wave 2

# pval = per-SNP alpha

#

#

power <- function( ncp1, ncp2, pval )

{

crit.val <- qnorm(pval/2,0,1)

power1 <- 1-pnorm( abs(crit.val), mean=sqrt(ncp1), sd=1 )

power2 <- 1-pnorm( abs(crit.val), mean=sqrt(ncp2), sd=1 )

power.joint <- 1-pnorm( abs(crit.val), mean=sqrt(ncp1 + ncp2), sd=1 )

c(power1, power2, power.joint)

}
