# Supplementary figures and images for: Multiethnic Genetic Association Studies Improve Power for Locus Discovery
Source: PLoS One. 2010 Sep 8;5(9):e12600. doi: 10.1371/journal.pone.0012600 (PMC2935880; doi:10.1371/journal.pone.0012600)

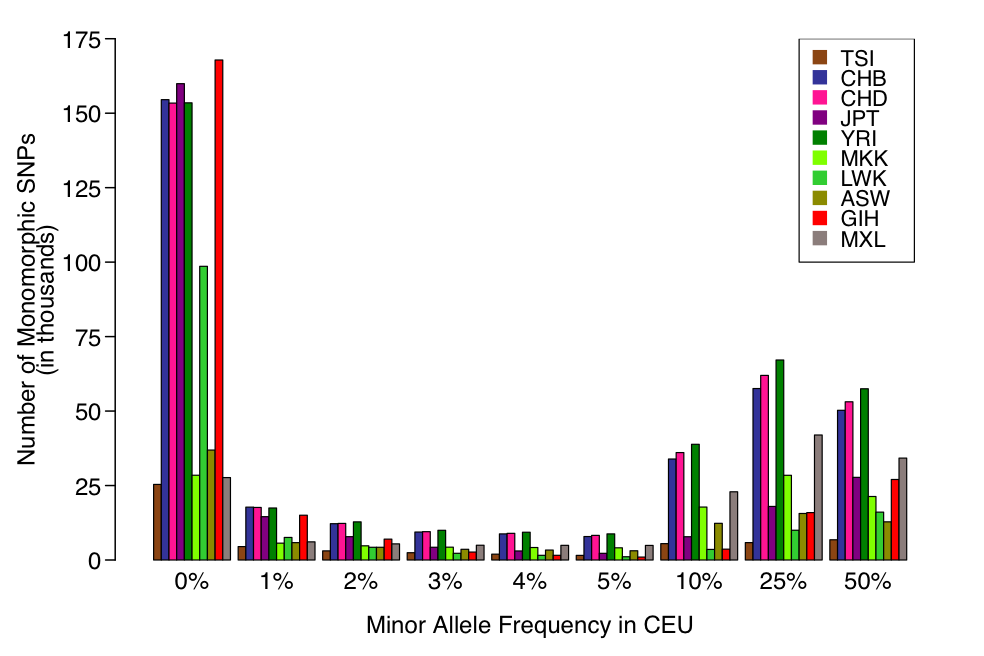

Supplement: Figure S1 — Number of monomorphic SNPs in the HapMap 3 population panels. The number of monomorphic SNPs in each population panel are displayed, stratified by minor allele frequency in CEU. (1.94 MB TIF) [file pone.0012600.s001.tif]

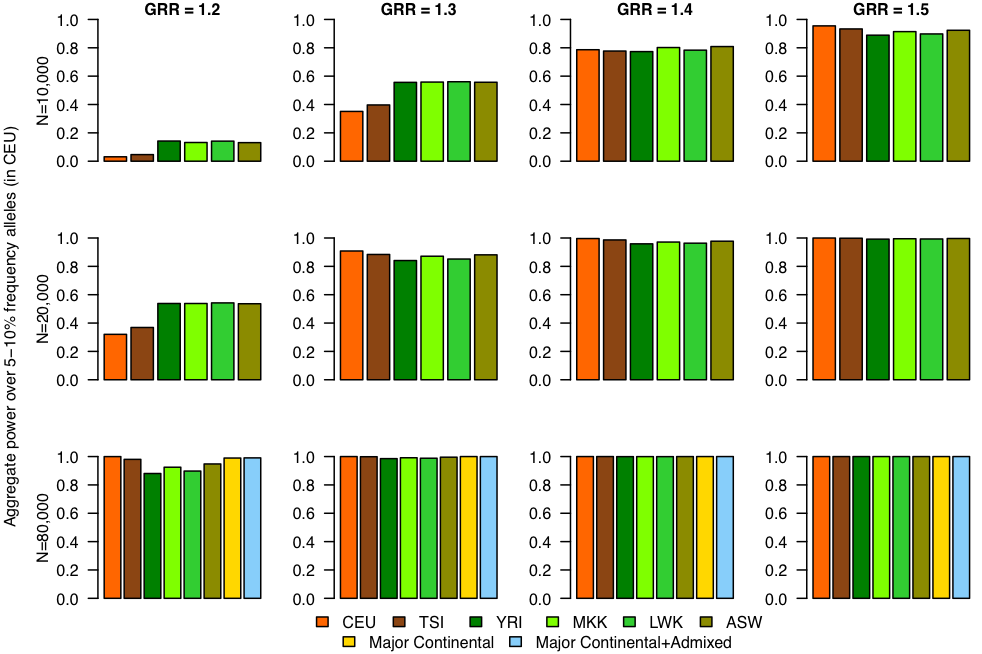

Supplement: Figure S2 — Power to detect an association for common alleles (5–10%) based on HapMap 3 data. The impact on power of switching to non-European samples in stage 2 of the GWAS is limited primarily to alleles of lower frequency in CEU. Testing non-European samples for alleles of common frequency in CEU yields a small (or no) increase in power. (1.93 MB TIF) [file pone.0012600.s002.tif]

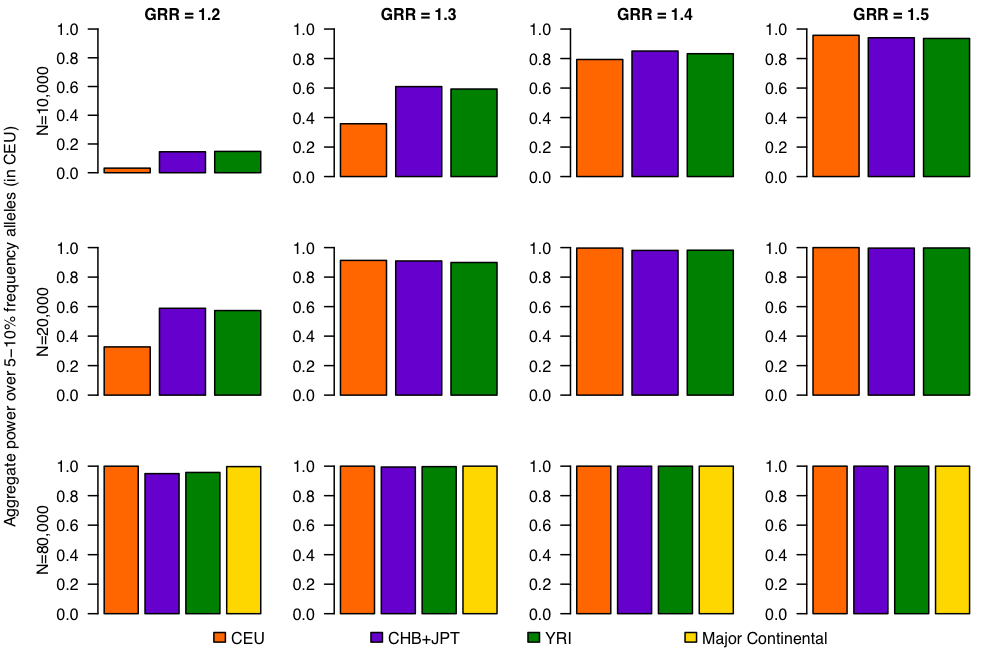

Supplement: Figure S3 — Power to detect an association for common alleles (5–10%) based on 1000 Genomes data. Consistent with our observations in the HapMap data, the improvement in power achieved by using non-European samples is limited to alleles of lower frequency in CEU. (1.93 MB TIF) [file pone.0012600.s003.tif]
